# Gut Microbioma Population: An Indicator Really Sensible to Any Change in Age, Diet, Metabolic Syndrome, and Life-Style

**DOI:** 10.1155/2014/901308

**Published:** 2014-06-04

**Authors:** Noce Annalisa, Tarantino Alessio, Tsague Djoutsop Claudette, Vasili Erald, De Lorenzo Antonino, Di Daniele Nicola

**Affiliations:** ^1^Division of Hypertension and Nephrology, Department of System Medicine, University of Rome Tor Vergata, Viale Oxford 81, 00133 Rome, Italy; ^2^Departement of Diagnostic and Molecular Imaging, Interventional Radiology and Radiation Therapy, University of Rome Tor Vergata, Viale Oxford 81, 00133 Rome, Italy; ^3^Department of Biomedicine and Prevention, Division of Clinical Nutrition and Nutrigenomic, University of Rome Tor Vergata, Viale Oxford 81, 00133 Rome, Italy

## Abstract

Obesity has become a pandemic threat in the latest 30 years. The trend of the prevalence of overweight and obesity has got an overall increase in every part of the world, regardless of ethnicity, life-style and social ties. High food intake, genetic, and sedentary have been related to obesity; it has been also hypothesized that gut microbiota could have an impact on the complex mechanism underlying the weight gain. This review aims to illustrate the actual literature about gut microbiota and its relation with obesity and to analyze the possible implications of factors such as diet and life-style onto the composition of gut microbiota, that can lead to overweight/obesity condition.

## 1. Introduction

### 1.1. Definition and Epidemiology of Obesity: Global and Regional Trends

The most widely parameter utilized in order to define overweight and obesity is Body Mass Index (BMI, defined as the weight in kilograms divided by the height in meters squared) [1]. This method is fast, simple, and inexpensive and is often preferred to more sophisticated tools due to its lower costs and availability [2].

Actually adult subjects with BMI values between 18.5 and 24.9 kg/m^2^ are considered normal, subjects with values between 25 and 29.9 kg/m^2^ are considered overweight, and those with BMI higher than 30 kg/m^2^ are considered obese, while adult subjects with BMI values lower than 18.5 are considered underweight [3].

Recently a group of researchers has proposed a new anthropometric classification by combining the values of BMI and fat mass percentage (FM%), obtained by DEXA (dual-energy X-ray absorptiometry). They identified a new syndrome, called “normal-weight obese (NWO) syndrome” [4] in women with normal weight and BMI whose fat mass is >30% of their total body weight. NWO women are characterized by early inflammation, related to their body fat mass, and their plasma proinflammatory cytokines concentration is significantly increased compared to nonobese women [5].

Due to obesity strict connection to several pathological conditions, like cardiovascular diseases, high blood pressure [6], type II diabetes [7], and many other chronic noncommunicable diseases (CNCD), its surveillance has been greatly improved in the latest years. In fact, it has been estimated that obesity causes more than 1 million deaths and 12 million life-years of ill health every year [8].

Several studies have proved that overweight and obesity are positively correlated with increased risk of mortality in 50-year-old nonsmokers [9]. A relevant higher prevalence in male subjects was also observed.

**Figure 1:**
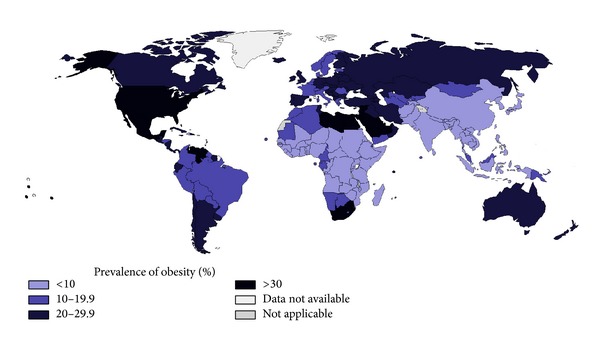
Worldwide Overall percentage of obesity prevalence (2008). Data source: WHO [1].

Furthermore, in the latest 20–30 years an increased global prevalence of obesity has been detected in almost every part of the world (Figure 1).

**Figure 2:**
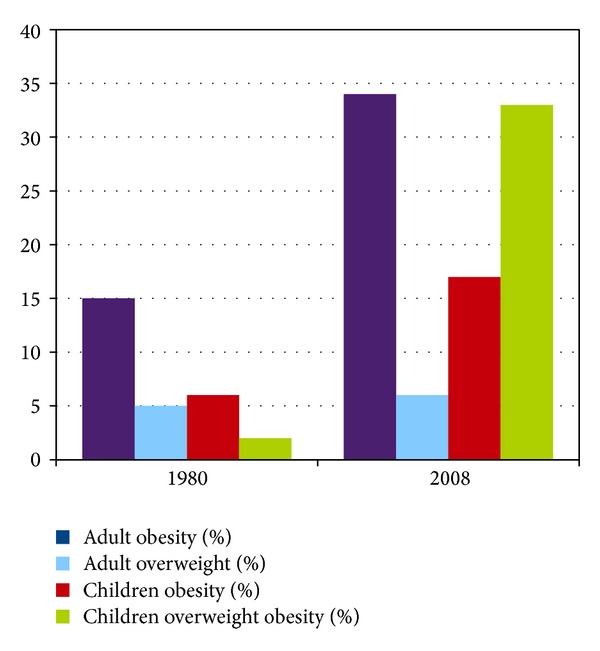
Prevalence's trend of overweight and obesity in USA population (1980–2008) [1].

Especially in USA, the obesity prevalence has greatly risen since 1980, when 15% of people were obese; in about 25 years prevalence has reached 34% [12]. In 2006 a study on US children aged from 2 to 19 years showed that 31.9% of them were at or above the 85th percentile of the* 2000 CDC BMI-for-age growth charts*, and 16.3% were at or above the 95th percentile of BMI for age. Considering that in 1980 children who were at or above 85th percentile were 5.5%, this study reveals a huge increase in US obesity and overweight rates, though since 2006 no relevant further increase of the BMI-stated obesity has been observed [12] (Figure 2).

Several European studies showed similar data. Despite the remarkable differences in life style, an overall increased prevalence of overweight and obesity has been detected, even in nations with a well-known low prevalence of high BMI values (mainly all the southern European states) [13].

**Figure 3:**
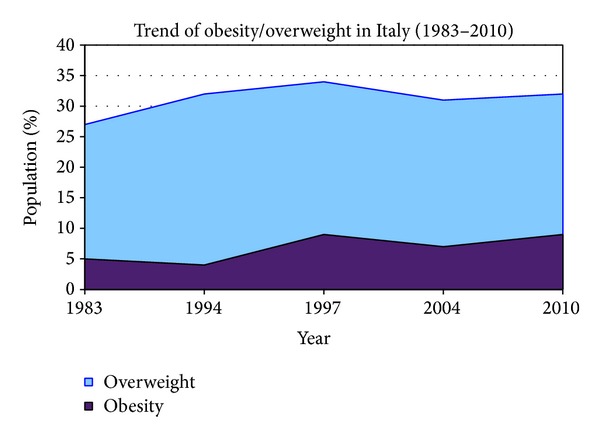
Trend of overweight and obesity in Italian population (1983–2010) [10, 11].

A group of researchers studied the obesity/overweight trend in Italy in the 1983–2010 period, finding an overall steady increase of prevalence of both conditions until 1997, with a substantial stabilization of the values since then (Figure 3) [14–17].

In particular, an overall increased prevalence of high BMI values has been observed in the richest countries, but the higher values have been recorded among the most disadvantaged social classes [18]. Although even in those subgroups data showed significant differences between various ethnic groups, a high prevalence of overweight/obesity has been recorded in all the groups, underlining the importance of some life-style factors (e.g., unhealthy dietary patterns, reduced physical activity, and the improved of motorized transports) [19, 20].

Some studies evidenced also a significant role for parent's educational level, especially mothers [21]. In the latest decades obesity and overweight have become epidemic even in Brazil-Russia-India-China (BRIC) [22] and developing countries, confirming the global increasing trend.

### 1.2. Obesity Etiopathogenesis

A well-defined etiopathogenesis is still far to be recognized; however, actually obesity is considered a complex, multifactorial condition resulting from the interaction between the environment, genetic and life-style factors [23].

Recent studies, however, showed a strongly genetic feature in the risk of becoming obese, in particular the homozygotes for mutation in human leptin receptor gene. In fact, the leptin receptor polymorphism has been established to be associated with obesity, especially in Caucasian populations [24]. Also the discovery of the fat mass and obesity-associated protein (FTO) gene showed the genetic role in the development of obesity and metabolic syndrome (MetS) [25].

Diet is also very important nowadays, because of the diffusion of food with high percentage of carbohydrate and highly refined oils causing an excess of energy intake and a relevant change of the most common dietary-patterns [20].

## 2. The Human Gut Microbiota

### 2.1. Definition

In recent years there has been a growing interest for the fact that humans are supraorganisms composed also by microbial cells [26]. Therefore, the humans carry two sets of genes, those encoded on their own genome and those encoded in microbiota genome (microbioma). The human cells genes are about 23,000 [27] and microbiota genes are about 3 million [28, 29]. Thus as superorganism, the humans inherit only 1% of their genes from their parents, and the remaining 99% is mainly acquired from the immediate environment when they are born, and in particular from their mothers' birth canal and breast milk [30, 31]. Importantly, all the genes in body, whether human or microbiome encoded, have the potential to have an impact on health status [32, 33].

The intestinal microbiota is considered as a symbiont that is part of organism since at birth that educates the immune system and contributes to the development of the intestinal vasculature and most probably of the nervous system (having a positive impact on cognition, memory, and other cerebral skills) [34].

A study of germ free mice (mice raised without exposure to any organism [GF]) had shown that the colonization with microbiota promotes vessel density in the small intestine [35]. The mechanisms that explain this process is the promotion of tissue factor (TF) glycosylation associated with its localization at the cell surface, the activation of coagulation proteases, and the phosphorylation of the TF cytoplasmic domain in the small intestine. This mechanism could favour the development of the immune system causing the maturation of microvasculature. Consequently the immune cells would reach the intestinal epithelium where they recognize bacterial antigens and establish an immune tolerance required to avoid a chronic inflammatory state [36, 37].

In the human body, the gut is the most densely colonized microbial community and consists of numerous species [38, 39]. Into it live trillions of microbes, composed approximately by 100 different kinds of bacteria, in a number of 10^10^ to 10^11^ bacteria per gram. The majority of microbiota is composed by strictly anaerobes, even if facultative anaerobes and aerobes are also present [40]. Although a huge number of different bacteria phyla have been identified, the overall bacterial population of human gut is determined basically by only 2 of them: Bacteroidetes and the Firmicutes, even if Proteobacteria, Verrucomicrobia, Actinobacteria, Fusobacteria, and Cyanobacteria are also present even if their role is unclear yet.

At present little is known about the development of the microbiota, even if it has been demonstrated that the microbiota starts in the early life to colonize human gut; this acknowledgement was acquired due to the observation of the similarities between the intestinal microbiota of infants and the vaginal microbiota of their mothers [41]. It was also recently stated that the composition of microbiota can be really variable even between people of the same ethnicity, nation, and life-style [42]. It has been speculated that the composition of gut microbiota comes from a complex interaction between several factors like dietary-pattern, ethnicity, genetic factors, and more. Some reports on twins also demonstrated that incidental environmental exposures play a major role in determining the distinctive characteristics of the microbial community, because despite considerable temporal variation, the distinct features of each baby's microbial community were recognizable for intervals of weeks to months [43].

The gut functions as a chemostat, a continuous culture system for microorganisms leave at a relative constant rate [44]. Approximately 1.5 kg of bacteria is resident in humans gut [33], and 50% of human faecal matter is bacterial cells. To maintain such a high population density, these bacteria need a lot of nutrients, which can come from food sources such as dietary fibres (which are otherwise indigestible to humans) [45, 46], from mucin and from cells sloughed from the gut, as well as drugs and other xenobiotic compounds [47, 48].

The host physiologic digestion (characterized by the pH of the gut and by the presence of bile acids) [49, 50] and the components of the innate immune response (such as defensins and immunoglobulin A), impose a selective pressure to determine the membership of the gut microbiota [51].

However, the microbiota composition is the result of the equilibrium between the ability to take advantage of the available nutrients and to withstand the forces removing it from the gut [52].

Recent studies demonstrated that ageing also has a major role in the transformation of microbiota; in fact the composition of microbial flora can be totally different between children, adults, and elderly. Microbioma can also change for pathological conditions (like chronic inflammatory guts diseases and metabolic disease such as type II diabetes) [53, 54].

### 2.2. Microbiota and Metabolism

Gut microbiota plays a key role in human metabolism; in fact, up to one-third of the small molecules in human blood can be derived from gut bacteria [55–57].

Metabolites produced by gut bacteria can enter the bloodstream by absorption, by enterohepatic circulation, or by impaired gut barrier function [58]. Some microbiota-derived metabolites can have a positive impact on the host, including those with anti-inflammatory [59], antioxidant [60], or pain relief activity [61], and those that regulate gut barrier function [62] as well as those acting as vitamins [63] or energy sources [64]. For example, butyrate, which is produced by bacterial fermentation of dietary fibres, can serve as energy source for colonocytes and can increase satiety [65, 66]. This compound is also effective in alleviating inflammation, reducing carcinogenesis, and mitigating oxidative stress [62, 67].

By contrast, other microbiota-derived metabolites are toxic to their host, including cytotoxins [68], genotoxins [69], and immunotoxins [70]. For example, lipopolysaccharide (LPS), an endotoxin released by gram-negative bacteria, can provoke an inflammatory response and thus aggravate inflammation-related chronic conditions such as insulin resistance [62, 70, 71].

The causal role of gut microbiota in the control of energy homeostasis was first demonstrated by comparative study between conventional (CV) mice and germ free (GF) mice fed with high-fat (HF) diet [72]. Whereas conventional mice gain weight as expected, the GF mice remained lean although their daily amount of food intake was dramatically increased [72], suggesting an impaired feeding efficiency. Body weight gain was greater when the germ free mice were colonized with the microbiota from obese rather than from a lean mouse [73]. Further studies demonstrated that the high-fat diet response is not only dependent on GF state but also dependent on type of HF-diet; it was stated that a diet with high content of sucrose had a greater lipogenic effect on CV mice than on GF mice [74, 75].

It was subsequently shown that obese patients were characterized by an intestinal microbiota dysbiosis where the proportion of the principal phyla (Firmicutes and Bacteroidetes) was changed in favour of an excess of Firmicutes when compared with lean controls [76]. This dysbiosis was reversed when the obese patients were submitted to caloric restrictions for several months. Interestingly, the obese leptin-deprived* ob*/*ob *mice showed similar intestinal dysbiosis [77] suggesting a common mechanism between mice and humans.

But others studies contradict with these previous reports with regard to the contribution of various bacterial microbiota composition to the development of obesity; in particular, the Bacteroides/Firmicutes ratio has been proved as insignificant [78, 79].

### 2.3. The Mechanisms Underlying Weight Gain

Due to the increase of the overweight status in the latest decades, the attention on the obesity condition increased in almost every field of research; obviously, also human gut and human gut microbiota were involved. The mutualistic nature of the host-bacterial relationship is underscored by mechanisms that underlie fat-storage phenotype. Our microbial partners have coevolved with us forging a symbiotic relationship, with mutual advantage. This relationship is established on nutrient sharing. Bäckhed et al. [80] stated in their study that one manifestation of this symbiotic relationship is microbial processing of components of the food intake and deposition the extracted energy in host fat depots. The ability to store energy was essential for ancient humans who did not have easy access to food. However, nowadays, because of the wide-spreading of large-portion, high-calorie diets, this “benefit” became a detriment.

Microbiota colonization increases glucose uptake in the host intestine and induces a substantial elevation in serum glucose and insulin, both of which stimulate hepatic lipogenesis through their activity on two transcription factors (ChREBP and SREBP-1c) [81].

Short-chain fatty acids, generated by microbial fermentation, also induce lipogenesis. Triglycerides exported by the liver into the circulation are taken up by adipocytes through a lipoprotein lipase- (LPL-)mediated process.

The microbiota suppresses intestinal epithelial expression of a circulating LPL inhibitor called fasting-induced adipose factor (Fiaf). Comparisons of GF, conventionalized wild-type and mice knocked-out for the Fiaf gene, established that Fiaf is a physiologically important regulator of LPL activity in vivo and a key modulator of the microbiota-induced increase in fat storage. However, it is reasonable to postulate that the caloric value varies between individuals due to their different gut microbiota composition, different intestinal transit time, and the fact that the microbiota influences human energy balance.

The humans are divided into the following: “high efficiency bioreactors” and “low efficiency bioreactors,” according to the ability to harvest energy from feeding and to promote storage. Relatively the first are involved into development of obesity while the last are significantly related to leanness.

The idea that individual variations in bioreactor efficiencies may be a significant variable in the energy balance equation is supported by several observations. First, individual variations in the composition of the microbiota occur and are influenced by host genotype. Second, small but chronic differences between energy intake and expenditure can produce major changes in body composition. Third, the microbiota is a substantial consumer of energy [81].

A recent study demonstrated that individuals on a “British diet” must ferment 50 to 65 g of hexose sugars daily to obtain the energy required to replace the 15 to 20 g of bacteria they excrete per day. In microbiota, there are species with larger capacities for processing dietary polysaccharides, such as Bacteroides, and they are more represented in lean subjects than in morbidly obese subjects. So, differences in the composition of the microbiota are associated with differences in gut bioreactor efficiency and predisposition of obesity [80].

As we adapt to changes of the environment, the gut microbiota adapts to qualitative and quantitative changes of our diet. Particularly, bacteria of the large intestine respond to changes in diet, especially to the type and quantity of dietary carbohydrate. One of the main consequences of increased carbohydrate intake is to decrease the pH of the gut lumen, significantly altering bacterial metabolism and commensal prevalence. The surplus of carbohydrates breakdown is channeled by components of the microbiota into energy storage. In fact, certain commensal species such as Firmicutes suppress circulating LPL inhibitors, thus inducing increased enzyme activity and energy accumulation as fat [82–85].

### 2.4. In Vitro/Animal Studies

A team lead by JI Gordon has studied the importance of gut microbiota as an environmental factor regulating fat storage and playing a role in obesity. In fact, the colonization of germ-free mice by mouse microbiota produces a massive increase in body fat (+60%) and insulin resistance. The association of Bacteroides thetaiotaomicron and Methanobrevibacter smithii increased the metabolic activity of germ-free colonized mice. Weight and fat increases in mice were not related to food consumption increase but, rather, to food conversion ratio increase (increase of weight/ingested food weight). When comparing offspring and mother microbiota in nongenetically obese mice, it was found that the offspring's microbiota reflected that of the mother [73].

Microbiota transplantation from either lean or obese mice into the gut of germ-free mice resulted in, respectively, less or more body fat, even when the caloric intake remained the same. Genetic and environmental factors influenced microbiota composition in mice. Microbiota is linked to obesity either as a cause (transfer of microbiota) or a consequence (obese genotype) [73].

Raoult recently found that a single adsorption of a Lactobacillus strain dramatically increased food conversion and weight increase in chickens. Taken together, the empirical data from agriculture and experimental data in laboratory animals showed that manipulating gut microbiota by antibiotic ingestion or by contamination with selected bacteria causes a significant weight gain. It is difficult to reject the hypothesis that antibiotics and probiotics may have the same effect in humans [86].

Bäckhed et al. report that, in contrast to mice with a gut microbiota, the germ-free (GF) mice are protected against the obesity that develops after consuming a high-fat and sugar-rich diet. Their persistently lean phenotype is associated with increased skeletal muscle and liver levels of phosphorylated AMP-activated protein kinase (AMPK) and its downstream targets involved in fatty acid oxidation (acetyl-CoA carboxylase; carnitine palmitoyltransferase). Moreover, GF knockout mice lacking fasting-induced adipose factor (Fiaf) are not protected from diet-induced obesity. Although GF* Fiaf*−*/*− animals exhibit similar levels of phosphorylated AMPK as wild-type mice in liver and gastrocnemius muscle, they have reduced expression of genes encoding the peroxisomal proliferator activated receptor coactivator (Pgc-1) and enzymes involved in fatty acid oxidation. Thus, GF animals are protected from diet induced obesity by two complementary but independent mechanisms that result in increased fatty acid metabolism [87–89].

The gut microbiota was also studied in order to assess a possible correlation with other chronic degenerative diseases like chronic inflammation, insulin resistance, type 2 diabetes mellitus, and atherosclerosis. Cani and Delzenne studied mice after a period of 2–4 weeks of high-fat diet for the purpose of finding any important relation between food intake, microbiota composition and development of obesity, and metabolic syndrome [90]. They found out a significant increase in plasma LPS and named this condition “metabolic endotoxemia,” because levels of circulating LPS were lower than those relevated during a canonic septic shock [91].

Furthermore it was observed that high LPS levels coming from a high-fat diet can be absorbed by gut epithelium inducing a low-grade inflammation that is strictly involved in the development of obesity. In this inflammatory process there is a reduction of the Bacteroides number and an increase of Firmicutes, a typical condition observed in obese subject [90].

### 2.5. In Vivo Studies

A remarkable number of researchers studied different populations (children, adult, elderly, lean, overweight, and obese), different even for ethnicity and social-economic conditions. The results obtained have demonstrated a dominant role of environment and others life-style related factors [92], although alterations in the human intestinal microbiota are also linked to pathologic conditions such as inflammatory bowel disease and irritable bowel syndrome [93].

Mueller et al. have showed that a well-defined microbiota population for any studied subgroup (such as age, socio-economic condition, life-style) cannot be identified [94].

The ways in which the guts microbe population influences obesity is not yet well-know; it was hypothesized that this can happen by several mechanisms, like increasing dietary energy harvest, promoting fat deposition, triggering systemic inflammation, perhaps modifying locomotor activity, and having central effects on satiety [95].

DiBaise and his group tried to find out a connection between obesity and gut microbiota suggesting that bacterial lipopolysaccharide derived from the intestinal microbiota may act as a triggering factor linking inflammation to high-fat diet-induced metabolic syndrome [96].

It has been suggested also that antibiotics and probiotics may be able to influence the BMI and weight gain due to their effects on microbiota population, causing an increase on fat and weight gain even without genetically predisposing factors or increased food intake [86].

#### 2.5.1. In Elderly Subjects

In old age there is a progressive change in bacteria population. Medical treatment, in particular antibiotics, affects competitiveness and colonization resistance. Furthermore, host physiology may be compromised in aged populations owing to a reduced taste and smell perception. For example, achlorhydria, which is often observed in elderly subjects, may result in reduced colonization resistance and lead to bacterial overgrowth of the stomach and the small intestine. In addition, elderly tends to deviate from usual dietary habits, which in turn may influence bacterial colonization [97, 98].

The microbiota also undergoes substantial changes at the extremes of life, in infants and older people. The core microbiota of elderly subjects was distinct from that previously established for younger adults, with a greater proportion of Bacteroides spp. and distinct abundance patterns of Clostridium groups. The fecal microbiota of the elderly shows temporal stability over limited time in the majority of subjects but is characterized by unusual phylum proportions and extreme variability [99].

In elderly persons bifidobacteria decrease or disappear, clostridia including* C. perfringens *significantly increase, and lactobacilli, streptococci, and enterobacteriaceae also increase.

Tomotari Mitsuoka retained that the abnormal flora are generally characterized by a remarkable increase in bacterial counts in the small intestine, by an increase of aerobes, mostly enterobacteriaceae and streptococci, by the reduction or disappearance of bifidobacteria, and/or often by the incidence of* C. perfringens. *This ecological evidence would suggest that bifidobacteria should exist in the large intestine for maintenance of health status and are far more important than lactobacilli as the beneficial intestinal bacteria throughout human life. In other words, the reduction or disappearance of bifidobacteria in the human intestine would indicate an “unhealthy” state [100–102].

#### 2.5.2. Adult Subjects

Turnbaugh et al. studied adult female monozygotic and dizygotic twin pairs concordant for leanness or obesity in order to assess the existence of a “core” human gut microbiome. In none of the 154 enrolled subjects was detectable a single bacterial phylotype by a set of abundant microbial lineages that we all share, proving that this hypothesis is wrong; hovewer, the authors found out an important connection in shared genes. This discovery gained major relevance when researchers assessed that those genes were greatly involved in a lot of very important metabolic functions. The authors compared this situation to an island, assesting that every person is an island with a unique gut microbioma, in which different species assemblages converge on shared core functions provided by distinctive components [103].

Collado et al. hypothesized that the gut microbiota composition differs in obese pregnant women compared with normal-weight pregnant women and it may be associated with weight gain during pregnancy. The deviations in gut microbiota composition during pregnancy could predispose to excessive energy storage resulting in higher birth weight. This is a vicious circle because overweight infants frequently become overweight adolescents and obese adults with a high risk of Western life-style diseases. These researches lead a comparative study onto a population of pregnant women: 18 overweight women and 36 normal-weight women (control group). They observed that microbial counts increased from the first to third trimester of pregnancy and high Bacteroides concentrations were associated with excessive weight gain over pregnancy. In addition, the differences in* Bifidobacterium *genus numbers between the first and third trimesters of pregnancy showed a correlation with normal weight gain over pregnancy, suggesting that* Bifidobacterium* counts were higher in women with lower weight gain over pregnancy [104].

A recent study reports that high-fat feeding is associated with lower concentrations of bifidobacteria [105]. High numbers of bifidobacteria may correlate positively with the normalization of inflammatory status and improved glucose tolerance and glucose-induced insulin secretion. Then, a low number of* Bifidobacterium *in overweight mothers may be associated with inflammatory processes.* Bifidobacterium* has thus an important effect on microbiota transfer from mothers to infants. It may be important to modify the microbiota of pregnant women to influence the first inoculum and the transfer of microbiota to the infant, because it may have a significant effect on the later health of the infant [104].

Patil et al. report a comparative and quantitative analysis of dominant gut microbiota of lean, normal-weight, obese, and surgically treated obese individuals of Indian origin. They observed that the bacteria of genus* Bacteroides* were prominent among the obese individuals. In addition, a remarkably high archaeal density was also noted in the obese group. On the contrary, the surgically treated obese individuals exhibited comparatively reduced* Bacteroides* and archaeal counts [106].

Fernandez-Raudales et al. compare, in a randomized double-blind study, the effects of consuming low glycinin soymilk (LGS), conventional soymilk (S), or bovine milk (M) on the intestinal microbiome in overweight and obese men. The participants were divided in three subgroups: consumed 500 mL daily of LGS, S, or M for 3 months. PCR analysis showed that the total bacteria increased in all treatments over time. Bacteroides-Prevotella and Lactobacillus increased in LGS and M, respectively.* Bifidobacterium* was significantly reduced in LGS. Bacterial diversity decreased for LGS, S, and M. Then, the consumption of the three beverages differentially altered the microbiota in overweight and obese men including a potentially beneficial alteration of the Firmicutes to Bacteroidetes ratio in both soymilk groups [107].

#### 2.5.3. In Babies and Young Subjects

By one year of age, the babies retained their uniqueness but they converged toward a profile characteristic of the adult gastrointestinal tract [43].

Tims et al. demonstrated that monozygotic twins have a more similar microbiota compared with unrelated subjects; the composition and temporal patterns of development of the intestinal microbiota in a pair of fraternal twins were strikingly similar, suggesting that genetic and environmental factors shape our gut microbiota in a reproducible way.

Then, lower BMI was associated with a more abundant network of primary fiber degraders, while a network of butyrate producers was more prominent in subjects with higher BMI. In the higher BMI subjects butyrate and valerate contents in the fecal matter were increased. These differences in microbial networks suggest a shift in fermentation patterns at the end of the colon, which could affect human energy homeostasis [108].

De Filippo et al. has examined two children population one coming from Europe and the other from rural Africa in a comparative study. Both in the Western world and in the developing countries diets rich in fat, protein, and sugar, together with reduced intake of unabsorbable fibers, are associated with a rapid increase in the incidence of noninfectious intestinal diseases. The potential protective effects of the diet on bowel disorders were first described by Burkitt who, working in Africa in the 1960s, noticed the remarkable absence of noninfectious colonic diseases in Africans consuming a traditional diet rich in fiber [109].

Vael et al. evaluate the intestinal flora in infants recruited prospectively from the general population at birth and followed up to the age of 3 years. High intestinal* Bacteroides fragilis* and low* Staphylococcus* concentrations in infants between 3 weeks from birth and 1 year were associated with a higher risk of obesity later in life. This study suggests that early differences in the composition of the intestinal microflora precede the development of obesity in children.

Modification of the intestinal microflora in infants might represent a new strategy for prevention and treatment of obesity [110].

Bervoets et al. [111] focused on the positive role of bifidobacteria [112, 113] and studied the composition of gut microbiota in obese and lean children. This study considered a population of 26 obese/overweight subjects (BMI 28.7 ± 6.5) and 27 lean subjects, in order to calculate the concentration of bacterial species mostly related with major digestive process (in particular:* Bacteroides*,* Bifidobacterium*,* Clostridium*,* Staphylococcus*, and* Lactobacillus*). The data showed an interesting elevated Firmicutes-to-Bacteroidetes ratio in the gut microbiota of obese adolescents and children. This result has been confirmed by other two studies of adolescent population [114, 115]. Kelishadi et al., in a randomized triple-masked controlled trial, studied the effects of synbiotics on inflammation markers in overweight/obese children and adolescents and reached very similar conclusions to previous studies [116].

## 3. Conclusion 

The relevance of gut microbiota has greatly increased since it was assessed its role into the development of overall prevalence of obesity. Actually, even if studies were lead to small sample size, all the authors agree that the composition of gut microbiota influences the development of overweight/obesity in children and the health status of adult subjects. However they disagree about which parameter (genetic, diet, life-style, and environment) is more important to shape it. Due to this controversy, we see that it is rather difficult to draw definite conclusions on the most important mechanism underlying weight gain.

Further studies should investigate the possibility to improve guts health status by preventing guts dysbiosis and characterize the weight gain process. Actually the results of several trials have been inconsistent with regard to the type of probiotic used, dosing and timing of agent selected, and the population likely to benefit.

## Abbreviation List

AMPC: Cyclic adenosine monophosphate
BMI: Body Mass Index
BRIC: Brazil-Russia-India-China
CNCD: Chronic noncommunicable diseases
ChrEBP: Carbohydrate-responsive element-binding protein
CKD: Chronic kidney disease
DEXA: Dual-energy X-ray absorptiometry
Fiaf: Fasting-induced adipose factor
FM%: Fat mass percentage
FTO: Fat mass and obesity associated gene
GF: Germ-free
LGS: Low glycinin soymilk
LPL: Lipoprotein lipase
NWO: Normal weight obese
PGC-1: Peroxisomal proliferator activated receptor coactivator
SREBP-1C: Sterol regulatory element-binding protein 1c.
